# Intergenerational diabetes and obesity—A cycle to break?

**DOI:** 10.1371/journal.pmed.1002415

**Published:** 2017-10-31

**Authors:** Ronald C. W. Ma, Barry M. Popkin

**Affiliations:** 1 Department of Medicine and Therapeutics, The Chinese University of Hong Kong, Prince of Wales Hospital, Shatin, New Territories, Hong Kong; 2 Hong Kong Institute of Diabetes and Obesity, The Chinese University of Hong Kong, Hong Kong; 3 Li Ka Shing Institute of Health Sciences, The Chinese University of Hong Kong, Hong Kong; 4 Department of Nutrition, Gillings School of Global Public Health, University of North Carolina at Chapel Hill, Chapel Hill, North Carolina, United States of America

## Abstract

Ronald Ma and Barry Popkin discuss the urgent need and challenges to reduce intergenerational transmission of obesity and diabetes.

## The diabetes apocalypse?

Globally, efforts to prevent diabetes and obesity have attained mixed success.

Although prevalence of adult-onset diabetes is declining in some countries, the prevalence of childhood obesity and young-onset diabetes, with risk of associated complications, continues to increase in many high- and lower-income countries [1]. More than 1. 9 billion children and adults are overweight and obese, and prevalence is rapidly rising, with the greatest increase found among children [2][3,4].

Ongoing efforts to promote lifestyle intervention have had limited impact on the obesity and diabetes epidemic because of several critical factors. One factor comprises early life effects, a new field that has emerged surrounding developmental origins of many noncommunicable diseases (NCDs). This is the impact of nutritional and other insults during pregnancy and the first 2 years of life on the long-term risk of diabetes. Beginning with pathbreaking work by Barker on the relationship between low birth weight infants and subsequent risk of many adverse cardiometabolic outcomes [5,6], this field expanded to include multigenerational studies in animals and humans to highlight some of the potential mechanisms that may mediate the effect of fetal and infant undernutrition and related insults, including, for example, epigenetic changes affecting pancreatic islets, adipose tissue, and vasculature [7]. A large number of human cohort studies have followed those exposed to nutritional and infection insults in the fetus and infancy through early adulthood and are even exploring intergenerational factors, all showing that early exposures, when interacting with obesogenic dietary and related factors, lead to much greater risks of NCDs [8–10]. Most importantly, the increased risk of obesity and diabetes in individuals exposed in utero to undernutrition appears to be most exacerbated in those whose metabolic capacities are “mismatched” when exposed to metabolic challenges posed by overnutrition later in life [11,12]. This pathway may be particularly relevant to low- and middle-income countries, including India and China, which have undergone rapid socioeconomic and nutritional transition, with both countries witnessing a dramatic increase in the prevalence of diabetes over recent decades [13].

A second critical factor involves risks affecting populations of non-European origin, including Hispanic, African, Middle Eastern, or Asian groups [14–18]. These populations appear to have greater adiposity in the visceral area; yet, at the same body mass index (BMI) as European origin adults, they have a greater likelihood of being susceptible to diabetes, which even led to many Asian countries proposing lower cutoffs for overweight and obesity [19]. There is no clear explanation of the causes for these differences, although they are the subject of much exploratory research [12]. However, more explorations do not explain the explosion of diabetes in China, a country with a relatively low prevalence of adults with BMIs over 30.

**Fig 1:**
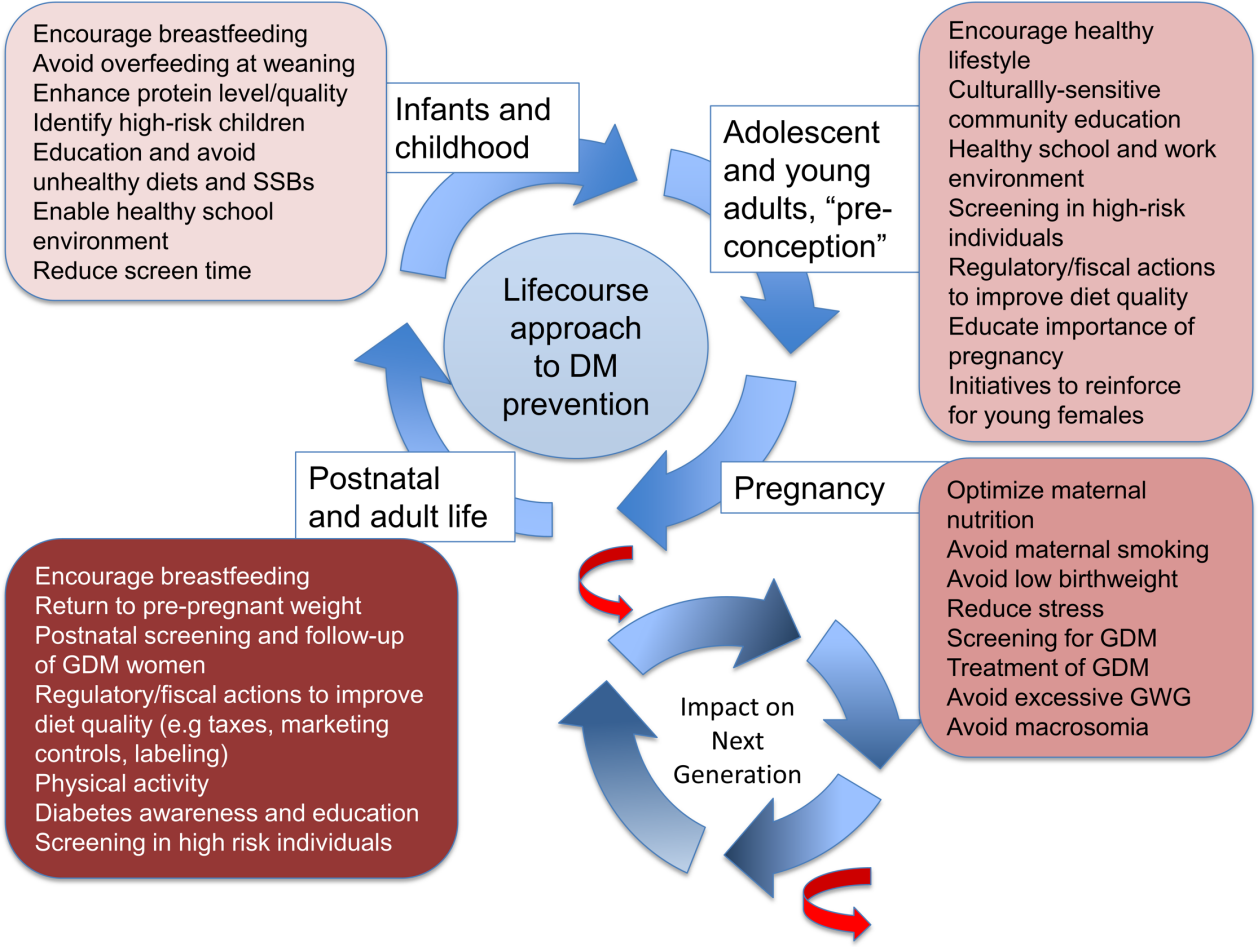
The intergenerational cycle of diabetes and obesity. Early life undernutrition, maternal diabetes, gestational diabetes, and maternal obesity are associated with increased risk of diabetes and obesity in the offspring, as well as younger age of onset of diabetes. This risk can therefore be transmitted to subsequent generations. Some potential interventions targeting different stages of the reproductive cycle and life course are highlighted.

Another aspect of developmental influences relates to maternal hyperglycemia, gestational diabetes mellitus (GDM), maternal obesity, and the resulting in utero overnutrition. Maternal obesity, GDM, and excessive gestational weight gain lead to macrosomia and excess adiposity in offspring and increase the risk of childhood obesity [20,21]. Furthermore, offspring to mothers with maternal hyperglycemia or maternal obesity are likely to develop diabetes at a younger age and may further propagate this intergenerational risk (Fig 1).

There is 1 last set of changes we are seeing across many countries. As overweight and obesity has increased globally [4], researchers find increased waist circumferences at the same BMI level over the last decade in many countries, which portends another unknown risk to diabetes [22].

## An informed approach to obesity and diabetes prevention

This intergenerational transmission of risk poses particular challenges to our current approach to obesity and diabetes prevention. Recent trials have highlighted that interventions initiated in pregnancy, such as increasing physical activity in overweight mothers, are likely to be too late to have an impact on pregnancy outcome [23–25]. Hence, there is a need to initiate interventions and dietary and physical activity changes in young women (and men) of reproductive age, who traditionally are least likely to come into contact with health services, placing particular challenges on outreach work. This need to engage these new at-risk groups, however, should not distract from ongoing efforts to reduce the pervasive obesogenic environment, including, but not limited to, reducing the intake of sugar-sweetened beverages (SSBs) and other energy-dense snacks and reducing sedentary lifestyle and screen time, especially among children [26]. A recent family-based study utilizing metabolomics has highlighted the important role of shared genetics and familial lifestyle between both parents and offspring in the pathogenesis of obesity [27]. Of note, prenatal and postnatal factors such as maternal smoking during pregnancy, gestational weight gain, duration of breastfeeding, and sleep duration during infancy can be associated with childhood obesity rates, which vary from 6% to 29%, suggesting that interventions that address these early life exposures may impact substantially on childhood obesity [28,29].

Several strategies have been explored over recent years, including restricting advertisement of unhealthy foods to children, front-of-pack labelling, improving school meals, using taxation to reduce the consumption of unhealthy food items, and providing different incentives to encourage intake of healthy foods or to encourage the production of healthier foods (Fig 1). However, the effectiveness and implementation of these remain challenging, and demonstration of impact on a national level remains lacking in most instances. Limited success stories are beginning to emerge, including the experience with taxation on SSBs in Mexico [30–32] and California, United States [33], in which, in both cases, the taxes reduced SSB intake and increased water purchases.

A keen sense of urgency is therefore needed in approaching the current conundrum. Not only is the global burden of diabetes giving rise to escalating healthcare expenditure that is threatening economic development of emerging economies, but the worsening epidemic of childhood obesity and young-onset diabetes and the increasing prevalence of GDM and diabetes affecting women of reproductive age are fuelling an evolving cycle of intergenerational risk transmission. Emerging data linking GDM with adverse neurocognitive development in offspring of mothers with GDM is particularly alarming [34], with maternal GDM potentially impacting on cognition and thereby further exacerbating the social disparity associated with NCDs [35]. Multisectoral efforts and intervention will be required to interrupt the current intergenerational transmission of risk [36] (Fig 1), but it is imperative that societies make this a top priority as the health and related developmental risks facing many countries are immense.

## Abbreviations

BMI: body mass index
GDM: gestational diabetes mellitus
NCD: noncommunicable disease
SSB: sugar-sweetened beverage
